# Fish Oil N-3 Fatty Acids Increase Adiponectin and Decrease Leptin Levels in Patients with Systemic Lupus Erythematosus

**DOI:** 10.3390/md13021071

**Published:** 2015-02-16

**Authors:** Marcell Alysson Batisti Lozovoy, Andréa Name Colado Simão, Helena Kaminami Morimoto, Bruna Miglioranza Scavuzzi, Tathiana Veiga Mayumi Iriyoda, Edna Maria Vissoci Reiche, Rubens Cecchini, Isaias Dichi

**Affiliations:** 1 Department of Pathology, Clinical Analysis and Toxicology, Rua Robert Koch, University of Londrina, Londrina, Paraná 86038-440, Brazil; E-Mails: marcell_lozovoy@hotmail.com (M.A.B.L.); deianame@yahoo.com.br (A.N.C.S.); helena@sercomtel.com.br (H.K.M.); reiche@sercomtel.com.br (E.M.V.R.); 2 Post Graduate Program in Health Sciences, Rua Robert Koch, University of Londrina, Londrina, Paraná 86038-440, Brazil; E-Mail: b_miglioranza@yahoo.com.br; 3 Post Graduate Program in Pathology, Rua Robert Koch, University of Londrina, Londrina, Paraná 86038-440, Brazil; E-Mail: tati_mvi@yahoo.com.br; 4 Laboratory of Pathophysiology of Free Radicals, Rua Robert Koch, University of Londrina, Londrina, Paraná 86038-440, Brazil; E-Mail: cecchini@uel.br; 5 Department of Internal Medicine, Rua Robert Koch, University of Londrina, Londrina, Paraná 86038-440, Brazil

**Keywords:** systemic lupus erythematosus, fish oil, adiponectin, leptin, cardiovascular risk

## Abstract

Cardiovascular disease (CVD) has emerged as an important cause of death in patients with systemic lupus erythematosus (SLE). Reduced adiponectin and elevated leptin levels may contribute to CVD in SLE patients. The purpose of this study was to verify the effects of fish oil (FO) on adiponectin and leptin in patients with SLE. Biochemical and disease activity analysis were performed. Patients with SLE were divided in two groups: patients who used fish oil for four months and patients who did not use fish oil. Patients with SLE who used FO had a significant decrease in SLE disease activity index (SLEDAI) score (*p* ˂ 0.023) in relation to baseline. SLE patients who used fish oil had increased adiponectin levels (*p* ˂ 0.026) and decreased leptin levels (*p* ˂ 0.024) compared to baseline values, whereas there were no differences in adiponectin and leptin levels in patients with SLE who did not use fish oil. In conclusion, the findings of increased serum adiponectin an decreased leptin levels after 120 days in the fish oil group, reinforce the importance of evaluating prospective studies of fish and fish oil fish ingestion on these adipokines in an attempt to decrease cardiovascular risk factors in patients with SLE.

## 1. Introduction

Systemic Lupus Erythematosus (SLE) is a systemic autoimmune disease characterized by multisystem organ involvement and by high titers of autoantibodies against several nuclear and cytoplasmic antigens [1].

Whereas the impact of infections and active disease on mortality has diminished dramatically over the years due to intensive treatment, cardiovascular disease (CVD) has emerged as one of the most important causes of death in these patients [2]. The incidence of myocardial infarction is five times as high in patients with lupus as in the general population, and in young women the age-specific incidence is increased by a factor of as much as 50 [3]. The pathogenesis of atherosclerosis is thought to be mediated, at least partially, by inflammation [4] and adipokines secreted in adipose tissue, in particular, adiponectin and leptin, have been implicated in atherogenesis and autoimmune diseases [5,6]. Several studies have reported the association of hypoadiponectinemia [7,8] and hyperleptinemia [9] with coronary heart disease.

The role played by adiponectin in SLE was verified through a peroxisome proliferator-activated receptor γ agonist rosiglitazone. This thiazolidone used clinically for the treatment of type 2 diabetes mellitus, reduced antibodies production, renal disease, and atherosclerosis in mouse models of SLE, and these beneficial effects were dependent on the induction of adiponectin, since these actions were not observed in lupus mice lacking adiponectin [10]. In addition, adiponectin deficiency in the context of a mouse model of lupus leads to more severe disease when compared to adiponectin-sufficient controls [11].

Meantime, experimental studies have revealed that the transcription factor peroxisome proliferator-activated receptor-γ (PPARγ) is perhaps the main mechanism by which *n-*3 fatty acids may increase adiponectin levels [12]. Therefore, it is conceivable to suggest that fish oil *n-*3 fatty acids besides their well- known anti-inflammatory and antithrombogenic effects could play a role in patients with SLE through their action on adiponectin. On the other hand, it has been reported that leptin-deficient mice are protected from T cell- and B cell-mediated inflammation in different disease models [13]. Studies about the effects of fish oil on leptin levels are scarce, but one study reported that a fish-rich diet decreased leptin levels in an African tribal population in Tanzania [14].

To the best of our knowledge, the present study is the first to report the effects of fish oil on adiponectin and leptin levels in patients with SLE. Our hypothesis was that fish oil *n-*3 fatty acids could increase adiponectin levels and decrease leptin levels in patients with SLE.

Therefore, the aim of the present study was to verify the effects of fish oil *n-*3 fatty acids on plasma adiponectin and leptin levels in patients with SLE.

## 2. Results and Discussion

### 2.1. Results

Demographic and clinical characteristics of the patients with SLE are shown in Table 1. There were no differences between the groups regarding age, gender, ethnicity, smoking and medications.

**Table 1:** Demographic, clinical and laboratory characteristics of patients with systemic lupus erythematosus.

Characteristic	Controls (*n* = 21)	FO (*n* = 41)	*p*-Value
Age (years)	42.5	43.0	NS
	(34.0–60.0)	(32.0–51.0)	
Gender *n* (%)			
Female	20	37	NS
Male	1	4	
Ethnicity *n* (%)			
Caucasian	17	32	NS
No Caucasian	4	9	
Smoking *n* (%)			
Yes	1	1	NS
No	20	40	
Prednisone			
Yes	20	39	NS
No	1	2	
Prednisone (mg/day)	10.0	10.0	NS
	(10.0–20.0)	(5.0–20.0)	
Antimalarials			
Yes	15	25	NS
No	6	16	
Current Immunosuppressive			
Yes	13	23	NS
No	8	18	

Mann-Whitney test. Data are median (25%–75%); FO, fish oil; NS, non significant.

Patients with SLE who used fish oil had significant decrease in systemic lupus erythematosus disease activity index (SLEDAI) (*p* ˂ 0.023) in relation to baseline values, although the median SLEDAI score 2 (0–10) showed that most patients had inactive or mildly active disease status at the beginning of the study. In contrast, other markers related to disease activity, such as C3, C4 and anti-dsDNA did not show significant differences between the groups (Table 2).

Table 3 shows the anthropometric and biochemical data of the patients. SLE patients using fish oil showed significant decrease in triacylglycerol (*p* ˂ 0.039) and increase in total cholesterol levels (*p* ˂ 0.026) compared to the control group. No differences were found in relation to body composition (WC and BMI), blood pressure, and HDL-cholesterol, LDL-cholesterol and glucose metabolism.

**Table 2:** Laboratory profile related to disease activity in patients with systemic lupus erythematosus using or not fish oil (FO).

Disease Activity Parameters	Control (*n* = 21)		*p*	FO (*n* = 41)		*p*
	T0	T120	(T0 *vs.* T120)	T0	T120	(T0 *vs.* T120)
C3 (μU/mL)	126	130	NS	110.0	107.0	NS
	(100–140)	(100–142)		(91.0–125.0)	(93.2–125.0)	
C4 (μU/mL)	20.8	21.5	NS	20.6	20.9	NS
	(13.1–25.7)	(12.5–126.0)		(14.1–25.2)	(15.5–28.8)	
Anti-dsDNA (titer)	0	0	NS	0	0	NS
	(0–10)	(0–10)		(0–20)	(0–5)	
SLEDAI	2	2	NS	2	0	0.0232
	(0–4)	(0–4)		(0–10)	(0–6)	

Data are median and interquartiles (25%–75%). Wilcoxon test was performed to verify changes from baseline (intra-group changes). Mann-Whitney test was performed to verify differences across treatment groups (inter-group changes) and to verify differences at the baseline (T0) between the groups; anti-dsDNA, anti-double-stranded DNA antibodies; SLEDAI, systemic lupus erythematosus disease activity index.

**Table 3:** Biochemical biomarkers of patients with systemic lupus erythematosus using or not fish oil (FO).

Biochemical Biomarkers	Control (*n* = 21)		Intra-Group Differences	FO (*n* = 41)		Intra-Group Differences
	T0	T120	*p*	T0	T120	*p*
			(T0 *vs.* T120)			(T0 *vs.* T120)
BMI (Kg/m^2^)	27.3	27.2	NS	25.6	25.5	NS
	(22.6–29.9)	(23.5–30.5)		(22.4–32.3)	(22.4–32.8)	
PAS (mmHg)	116.5	118.5	NS	114.0	120.0	NS
	(107.0–127.5)	(107.5–128.0)		(109.5–126.0)	(105.8–135.0)	
PAD (mmHg)	74.0	77.5	NS	76.0	74.0	NS
	(70.0–87.0)	(72.8–87.5)		(68.0–86.5)	(69.0–81.5)	
Triacil (mg/dL) *	103.0	120.0	NS	112.0	95.5	0.039
	(78.0–153.0)	(80.0–150.0)		(69.0–143.0)	(79.3–129.8)	
Chol (mg/dL) *	201.0	220.0	NS	193.0	205.0	0.026
	(178.0–225.0)	(180.0–250.0)		(162.0–216.0)	(181.8–232.3)	
HDL (mg/dL)	59.0	55.0	NS	55.0	53.5	NS
	(44.0–69.0)	(42.0–70.0)		(42.0–66.0)	(44.0–67.3)	
LDL (mg/dL)	116.0	120.0	NS	107.0	129.5	NS
	(103.0–145.0)	(150.0)		(91.0–127.5)	(102.8–144.5)	
Glucose (mg/dL)	94.5	95.0	NS	83.0	86.0	NS
	(78.5–103.8)	(82.5–107.5)		(80.0–93.0)	(82.0–92.0)	
Insulin (μU/mL)	9.55	9.90	NS	11.5	12.3	NS
	(6.60–12.6)	(7.10–13.2)		(6.0–16.8)	(6.5–12.9)	
HOMA-IR	1.89	2.02	NS	2.47	2.26	NS
	(1.59–3.03)	(1.68–3.38)		(1.28–3.63)	(1.59–3.17)	

Data are median and interquartiles (25%–75%). Wilcoxon test was performed to verify changes from baseline (intra-group changes). Mann-Whitney test was performed to verify differences across treatment groups (* inter-group changes) and to verify differences at the baseline (T0) between the groups. BMI, body mass index; SBP, systolic blood pressure CRP, *C*-reactive protein; DBP, diastolic blood pressure; Triacil, triacylglycerol; Chol, total cholesterol; HDL, high-density lipoprotein; LDL, low-density lipoprotein; HOMA-IR, homeostasis model of assessment insulin resistance.

**Figure 1:**
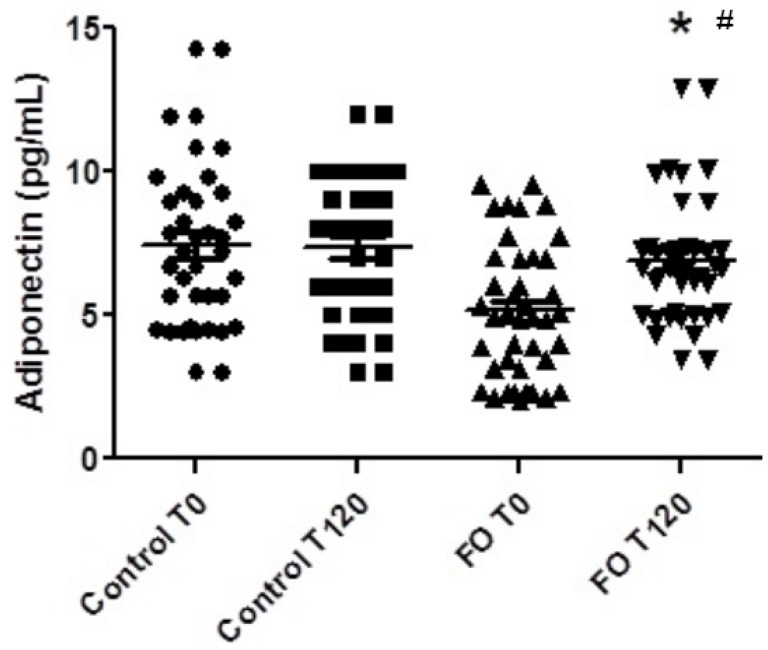
Plasma adiponectin levels in patients with systemic lupus erythematosus submitted or not to treatment with *n-*3 fish oil fatty acids for 120 days. Control T0, control group at the beginning of the study; Control T120, control group after 120 days; FO T0, fish oil group at the beginning of the study; FO T120, fish oil group after 120 days. # *p* < 0.05, FO T0 *vs.* FO T120. * *p* < 0.05, inter-group changes.

**Figure 2:**
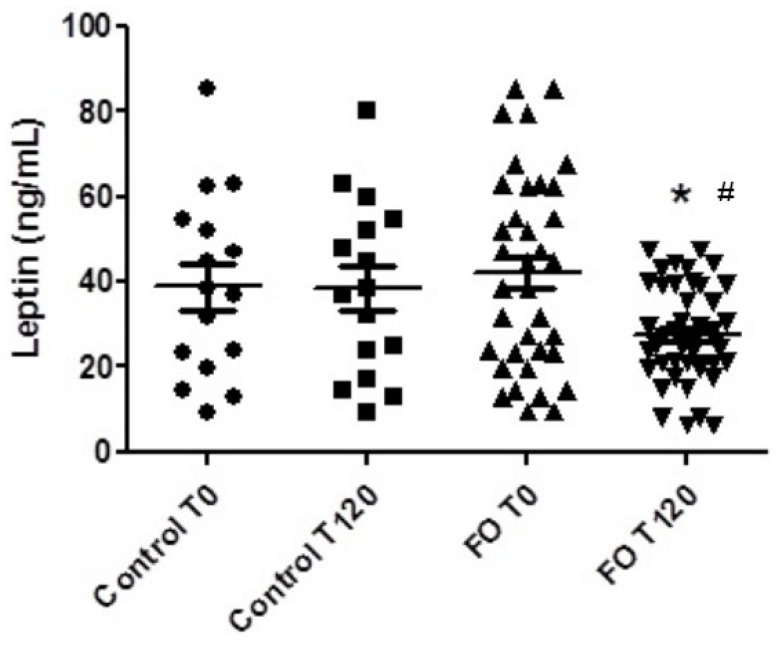
Plasma leptin levels in patients with systemic lupus erythematosus submitted or not to treatment with *n-*3 fish oil fatty acids for 120 days. Control T0, control group at the beginning of the study; Control T120, control group after 120 days; FO T0, fish oil group at the beginning of the study; FO T120, fish oil group after 120 days. # *p* < 0.05, FO T0 *vs.* FO T120. * *p* < 0.05, inter-group changes.

SLE patients who used fish oil had increased adiponectin levels (*p* ˂ 0.026) and decreased leptin levels (*p* ˂ 0.024) after four months compared to baseline values (Figure 1 and Figure 2, respectively), whereas there were no differences in adiponectin and leptin levels in patients with SLE who did not use fish oil. Additionally, inter-group differences were not observed.

### 2.2. Discussion

The main findings of the present study were the increase in plasma adiponectin and decrease in plasma leptin levels in patients with SLE who ingested fish oil. In addition, patients who ingested fish oil had a decrease in triacylglycerol and an increase in total cholesterol levels.

The decrease in triacylglycerol level is the most expected action of *n-*3 fatty acids [15]. In the present study, triacylglycerol levels decreased after fish oil consumption after 120 days. The strongest evidence to explain the decrease in triacylglycerol concentration is the reduced hepatic lipogenesis or, more specifically, reduced hepatic production and secretion of VLDL [16]. This effect is related to the direct inhibition of diacylglicerol acetyl-transferase and phosphatic acid phosphohydrolases, two key enzymes involved in triacylglycerol synthesis in the liver [17]. A decrease in serum triacylglycerol generally occurs in the ranges of 3–4 g/d [16] and is an expected and beneficial effect of fish oil on blood lipid profiles [17]. Balk *et al.* [18] pooled the results of 21 trials involving about 8000 patients taking *n-*3 fatty acids and, similarly to our data, there were significant decreases in triacylglycerol, however, differently from the present study, those authors found significant increases in LDL cholesterol levels, while there were no effects of fish oil supplementation on total cholesterol concentration. Meanwhile, there were no changes in HDL cholesterol levels in both studies.

Although non-significant, LDL increase seems to explain elevated cholesterol levels found in this study. Several reports have verified an increase in LDL and total cholesterol when fish oil is used at doses of greater than 3 g/d [16]. The assumption is that an increase in the concentration of LDL cholesterol by fish oil results directly from the enhanced conversion of very-low-density lipoprotein to LDL. Thus, *n-*3 fatty acids result in fewer VLDL particles secreted from the liver into the peripheral circulation and the VLDL particles secreted are rapidly converted to LDL particles [19]. In a recent study in patients with metabolic syndrome, our group verified that patients who used fish oil for three months presented significant decrease in triacylglycerol values and significant increase in total cholesterol and LDL cholesterol in relation to baseline values [20].

Differently from obese and metabolic syndrome patients who showed decreased adiponectin levels [21], several studies with patients with SLE have shown an increase in plasma adiponectin levels [22,23,24]. This increase has been interpreted in patients with SLE as a counter-regulatory and protective mechanism in response to chronic inflammation [25], or to metabolic disarrangements related to insulin resistance [24]. Sada *et al.* [22] and Chung *et al.* [24] showed higher adiponectin levels in patients with SLE, even when an inverse association between adiponectin levels and insulin resistance was shown. However, similarly to other studies [25,26,27], in the present study we did not observe an increase in plasma adiponectin levels. Of note, Rovin *et al.* [28] only reported increased adiponectin levels in patients with renal SLE compared to healthy controls and patients with nonrenal SLE. In the current study, patients did not have laboratorial signs of renal dysfunction or proteinuria.

The precise role of corticosteroid in adiponectin levels is controversial. Adiponectin levels were independent of corticosteroid therapy in patients with SLE in some studies [22,23], whereas another study has shown a positive association between adiponectin and corticosteroid therapy [25]. In the present study, corticosteroid therapy seems to have no influence on adiponectin values as both groups began the work with similar and low doses. In addition, corticosteroid dose did not change in both groups at the end of the study.

*N-*3 fatty acids have shown to increase adiponectin levels in both experimental [29,30] and human studies [29,31,32]. Itoh *et al.* [29] reported that eicosapentaenoic acid (EPA 1.8 g/d) increases adiponectin secretion possibly through the improvement of the inflammatory changes in obese adipose tissue in rodent models of obesity and human obese subjects. Lara *et al.* [31] also verified a trend towards an increase in plasma adiponectin, independent of weight change with 125 g/d of salmon consumption (2.4 g/d *n-*3 fatty acids) in non-obese healthy subjects. Recently, our group also showed an increase in serum adiponectin, nitric oxide and blood pressure after fish oil ingestion in patients with metabolic syndrome [32]. Experimental studies have revealed that the transcription factor peroxime proliferator-activated receptor-γ (PPARγ) is perhaps the main mechanism by which *n-*3 fatty acids may increase adiponectin levels [10]. Of note, *n-*3 fatty acids act like the thiazolidinediones, which are PPARγ agonists and are used in the treatment of insulin-resistant and diabetic subjects [33]. It is likely that increase in adiponectin levels with fish oil found in the present study is due to the aforementioned mechanism. In addition, both fish oil intake [34] and increased adiponectin levels [35] facilitate the uptake of early apoptotic cells by macrophages, an essential feature of immune system function. Therefore, it is conceivable to suggest that fish oil action in this case may be mediated by increasing adiponectin levels.

On the other hand, our data are in agreement with several studies, which have shown increased leptin levels in patients with SLE [22,24,25,26,27,36]. However, in other studies, elevated leptin levels have not been found [13,23]. In human studies, increased leptin levels have been suggested to play a role regarding cardiovascular disease risk factor in SLE. This hypothesis was reinforced with the findings of inverse association between leptin with both insulin resistance and lupus activity [24,26]. In addition, McMahon *et al.* [27] showed that high leptin levels contributed to 2.8 fold increased odds for the presence of atherosclerosis in women with SLE. Our data of decreasing leptin levels with fish oil supplementation expand the knowledge propitiated by Winniki’s *et al.* [14] study in a Tanzanian population ingesting a rich-fish diet, but now in patients with SLE.

Production of TNF-α has been shown to decrease adiponectin and increase leptin levels both in metabolic and inflammatory conditions [37]. Thus, it is conceivable to suggest that a common pathophysiological effect represented by suppression of TNF-α can be obtained through fish oil supplements [38].

The following limitations have to be considered in this study. First, there were a small number of participants. Second, this is a population with low disease activity shown by SLEDAI, and therefore, our findings may not be applicable to patients with very active disease. Nevertheless, the present study also has several strengths. First, to our knowledge, this is the first study to evaluate adiponectin and leptin levels in patients with SLE using fish oil. Second, we rigorously tried to assure that the patients did not take any drug or presented any disease, which could interfere with the results. In addition, statistical analysis between the groups were not significantly different in the patients using antihypertensive drugs, such as angiotensin-converting enzyme inhibitors, which may elevate plasma adiponectin levels [39]. Third, both groups were similar in relation to all parameters evaluated at the beginning of the study.

## 3. Experimental Section

### 3.1. Subjects

The study included 62 patients (57 females and 5 males) with SLE who were selected at the ambulatory of Rheumatology of the University Hospital of Londrina, Paraná, Brazil, to participate in the study. Systemic Lupus Erythematosus was diagnosed using the American College of Rheumatology (ACR) 1997 revised criteria [40]. Patients with SLE were divided in two groups: patients who used fish oil for four months and patients who did not use fish oil (controls). Information on lifestyle factors and medical history were obtained at clinical evaluation. Disease duration, organ involvement, values of C3 and C4 complement, anti-double-stranded DNA antibodies (anti-dsDNA), SLEDAI score, and non-steroid anti-inflammatory drugs, corticosteroids, antimalarial, oral contraceptives, and antihypertensive medications were recorded for each patient. Prednisone was the only kind of corticosteroids the patients were taking at the time of inclusion, thus prednisone-equivalent calculation was not required. They had been taking the same prednisone dose at least for the past 4 months. None of the subjects was receiving a specific diet. The individuals of both groups did not drink alcohol regularly. None of the participants in the study presented thyroid, renal, hepatic, gastrointestinal, oncological or other auto-immune disease, and none were receiving estrogen replacement therapy, lipid-lowering drugs, drugs for hyperglycemia, fish oil or antioxidant supplements. No patient presented proteinuria or increased creatinine levels at the beginning of the study. Patients who were taking antihypertensive drugs were not excluded and were allowed to continue taking the same dose of the drugs. This study was conducted according to the guidelines laid down in the Declaration of Helsinki and the Ethical Committee of the University of Londrina, Paraná, Brazil approved all procedures involving human subjects and patients. Written informed consent was obtained from all patients.

### 3.2. Study Design

Patients were assigned to one of two groups after stratification by sex, age, ethnicity, body mass index (BMI), and waist circumference (WC). The first group (control group, *n* = 21) was only directed to maintain their usual diet and the second group (fish oil group, *n* = 41) received 3 g/d of fish oil *n-*3 fatty acids (10 capsules). Each fish oil capsule contained 180 mg of eicosapentaenoic acid (EPA) and 120 mg of docosahexaenoic acid (DHA) originated from sardines. The capsules were given at breakfast, lunch, and dinner. The subjects were recommended to avoid resting after meals to avoid unpleasant effects. Both groups were evaluated at the beginning of the study and after 120 days. Opção Fênix manufacturers of pharmaceutical products provided fish oil capsules.

Anthropometric measurements and biochemical parameters were assessed at the beginning of the study and after 120 days.

### 3.3. Steps Taken to Optimize Compliance

Various measures were taken to optimize and assess patient compliance [41]. Before each trial began, it was assured that the patients understood that they could be allocated to any group. Boxes of fish oil capsules were handled out at the initial interview and at the two later visits. The subjects were asked to return the boxes at each visit so that the number of capsules taken could be estimated by questioning the patients and counting the remaining capsules.

### 3.4. Anthropometric and Blood Pressure Measurements

Body weight was measured to the nearest 0.1 kg by using an electronic scale, with individuals wearing light clothing, but no shoes, in the morning. Height was measured to the nearest 0.1 cm by using a stadiometer. Body mass index was calculated as weight (kg) divided by height (m) squared. Waist circumference (WC) was measured with a soft tape on standing subjects midway between the lowest rib and the iliac crest. Three blood pressure measurements taken with a minute interval between them after the subject had been seated were recorded. The mean of these measurements was used in the analysis [42].

### 3.5. Biochemical and Immunological Biomarkers

After fasting for 12 h, the patients underwent the following laboratory blood analysis: glucose, total cholesterol, HDL cholesterol, LDL cholesterol, and triacylglycerol, evaluated by a biochemical auto-analyzer (Dimension Dade AR Dade Behring, Deerfield, IL, USA), using Dade Behring^®^ kits; plasma insulin levels were determined by MEIA (AxSYM, Abbott Laboratory, Abbott Park, IL, USA). The homeostasis model assessment (HOMA) was used as a surrogate measurement of insulin sensitivity [43]. HOMA for insulin resistance = insulin fasting (μU/mL) × glucose fasting (mmol/L)/22.5. Insulin resistance was considered when HOMA ≥ 2.114.

Serum complement factors C3 and C4 levels were measured using a nephelometric assay (Behring Nephelometer II, Dade Behring, Marburg, Germany). Anti-double-stranded DNA (anti-dsDNA) antibodies were determined by immunofluorescence using Crithidia lucilliae kinetoplast assay (Obelis S/A, Brussels, Belgium). Disease activity was determined by using SLEDAI score [44].

Adiponectin and leptin levels were measured by a sandwich enzyme-linked immunosorbent assay (ELISA) using a commercial immunoassay ELISA (Ready-Set Go! Set, e-Bioscience, San Diego, CA, USA).

### 3.6. Statistical Analysis

The distribution of gender, ethnicity, smoking, and prednisone use were analyzed by a Fisher Exact test. Antimalarial and current immunosuppressive therapy were analyzed by chi-square test. Mann-Whitney test was performed to compare differences between parameters of groups at baseline and differences across treatment groups (inter-group changes). Wilcoxon matched pairs test was performed to verify changes from baseline (intra-group changes). Data are presented as median (25%–75%). Significance was set at *p*-value < 0.05. A statistical analysis program (Graph Pad Prism version 4.0, GraphPad Software Inc., California, CA, USA) was used for evaluations.

## 4. Conclusions

The findings of increased serum adiponectin an decreased leptin levels after 120 days in the fish oil group, reinforce the importance of evaluating prospective studies of fish and fish oil fish ingestion on these adipokines in an attempt to decrease cardiovascular risk factors in patients with systemic lupus erythematosus.
